# The Role of Ferroptosis in Cardiovascular Disease and Its Therapeutic Significance

**DOI:** 10.3389/fcvm.2021.733229

**Published:** 2021-10-26

**Authors:** Zhenzhen Chen, Youyou Yan, Chao Qi, Jia Liu, Longbo Li, Junnan Wang

**Affiliations:** Department of Cardiology, Second Hospital of Jilin University, Changchun, China

**Keywords:** ferroptosis, reactive oxygen species, lipid peroxidation, autophagy, cardiovascular diseases

## Abstract

Cardiovascular diseases (CVDs) are the leading cause of deaths worldwide with regulated cell death playing an important role in cardiac pathophysiology. However, the classical mode of cell death cannot fully explain the occurrence and development of heart disease. In recent years, much research has been performed on ferroptosis, a new type of cell death that causes cell damage and contributes to the development of atherosclerosis, myocardial infarction, heart failure, and other diseases. In this review, we discuss the role of different organelles in ferroptosis and also focus on the relationship between autophagy and ferroptosis. Additionally, we describe the specific mechanism by which ferroptosis contributes to the development of CVD. Finally, we summarize the current research on ferroptosis-related pathway inhibitors and the applications of clinically beneficial cardiovascular drugs.

## Introduction

The term “cardiovascular disease” (CVD) refers to a group of diseases that include heart disease, vessel disease, heart attack, stroke, heart failure, arrhythmia, and heart valve disorders. The prevalence of CVD-associated morbidity and mortality are increasing each year with the associated healthcare burden being a leading problem worldwide. Therefore, the prevention, treatment, and prognosis of CVDs have emerged as key areas of medical research in recent years (1–3). Previous studies have shown that various forms of regulated cell death (RCD), including apoptosis, pyrolysis, and necrosis, can destroy the structure of the heart and blood vessels and disrupt their physiological functions, thereby promoting the development of CVDs (4). Recent studies have reported that emricasan (an inhibitor of apoptosis), necrostatin-1 (an inhibitor of necrosis), or 3 -methyladenine (3-MA; an inhibitor of autophagy) fail to improve the survival of mice exposed to doxorubicin (DOX)-induced cardiotoxicity, whereas ferrostatin-1 (a ferroptosis inhibitor) is able to reduce mortality of these mice (5). Further studies found that lipid peroxidation occurred in the mitochondrial membrane. Although this occurs in multiple regulated death modalities, the use of a Fer-1 was found to ameliorate the cellular damage (5). This suggests that ferroptosis, which is a novel type of cell death, may play an important role in the development of CVD.

Ferroptosis was first proposed by Dixon et al. (6). Inhibition of cystine uptake by erastin induces oxidative death and results in the accumulation of lipid reactive oxygen species (ROS), smaller mitochondria, and increased membrane density. This occurs without typical apoptotic features, such as cell shrinkage, nuclear fragmentation, and apoptotic body formation, without necrotic morphological features, such as cytoplasmic and organelle swelling, and without typical features of autophagy, such as membrane-encapsulated vesicle formation (6). Deferoxamine (DFO) can inhibit this type of cell death (6). Another compound, RSL3, has also been shown to induce a similar cell death process (7). Researchers have found that ferroptosis is widely involved in the pathophysiology of a variety of conditions, including nervous system diseases, ischemia/reperfusion injury, kidney injury, and blood disorders (8). Changes in ROS levels are significantly associated with ferroptosis; this association is of concern as increased ROS levels are an important pathophysiological basis for the development of various CVDs. Therefore, an insight into the mechanism underlying ferroptosis-induced cell damage is crucial for a detailed understanding of CVD pathogenesis.

## Basic Characteristics of Ferroptosis

The biochemical aspects of ferroptosis manifest as the accumulation of lipid peroxides and increased Fe^2+^ levels. Mitochondrial morphology relative to ferroptosis is characterized by smaller mitochondria, increased membrane density, reduced or disappearance of mitochondrial cristae, and ruptured outer membranes (8). However, while iron can promote lipid peroxidation and ferroptosis by generating free radicals through the Fenton reaction, it is not essential for ferroptosis. For instance, it was recently determined that metallic copper ions can induce cell death by reducing glutathione (GSH) levels via the inhibition of glutamate-cysteine ligase (GCL) activity (9). That noted, the effect of copper ions on ferroptosis requires further study. Accumulated lipid peroxides generally exert their toxic effects as a key factor in ferroptosis induction through two mechanisms, by destroying membrane integrity and by altering the fluidity and permeability of the cell membrane. As highly reactive molecules, lipid peroxides interact with Fe^2+^ to further generate ROS. The lipid degradation products 4-hydroxynonenal (4-HNE), malondialdehyde (MDA), and acrolein form covalent adducts with proteins, DNA, and phospholipids, thereby altering the structure and function of the proteins and nucleic acids and inducing the development of a range of diseases (10, 11).

## Role of Organelles in the Regulation of Ferroptosis

Mitochondria, lysosomes, and endoplasmic reticulum play significant roles in the regulation of ferroptosis. Mitochondria provide energy for cells through oxidative phosphorylation and play a key role in cell metabolism and signal transduction regulation (12), with most free radicals in the body being produced in mitochondria. The morphological changes associated with ferroptosis mainly manifest as small mitochondrial and the reduction or disappearance of cristae (8, 13). The activation of mitochondrial voltage-dependent anion channels (VDACs) and siderofexin-1 (SFXN1) can significantly promote ferroptosis (14, 15), suggesting mitochondria are an important part of ferroptosis. However, there have been some recent studies that challenge this concept. For instance, Gao et al. found that inhibition of the mitochondrial tricarboxylic acid cycle (TCA) or electron transport chain (ETC) can reduce cysteine deficiency-induced ferroptosis whereas ferroptosis induced by the inhibition of glutathione peroxidase 4 (GPX4) with Ras-selective lethal small molecule 3 (RLS3) has no clear mitochondrial involvement (16). This may be explained by the fact that lipid ROS can be rapidly amplified through the Fenton reaction and subsequently induce ferroptosis. In contrast, another study found that the mitochondria-targeted ROS scavenger mitoquinone (MitoQ) attenuates RLS3-induced lipid peroxidation, mitochondrial ROS production, and loss of mitochondrial membrane potential in neuronal HT22 cells and mouse embryonic fibroblasts (17). Thus, the role of mitochondria in ferroptosis may be cell-type dependent and change under different conditions. As mitochondria participate in various aspects of redox reactions, understanding the role mitochondria play in ferroptosis may provide important cues for the direction of future research.

Ferroptosis is considered an autophagy-dependent mode of cell death (18, 19). Lysosomes are the main organelles for autophagic degradation and thus play a key role in ferroptosis. Torii et al. found that the lysosomal inhibitor Baf-A1 reduces ferroptosis of tumor cells (20). It has been previously suggested that the autophagy-lysosome pathway inhibits CVD progression (21, 22). However, most of the current studies on the relationship between the autophagy-lysosome pathway and ferroptosis have focused on tumor cells with the findings suggesting that autophagy promotes ferroptosis. Accordingly, the autophagy-lysosome pathway need to be further studied in the backdrop of CVDs.

In addition to mitochondria and lysosomes, we found that the endoplasmic reticulum may also be involved in ferroptosis. Cells make adaptive changes when exposed to hypoxia, oxidative stress, and toxic substances that cause protein misfolding, unfolded protein aggregation, and dysregulation of calcium homeostasis in the lumen of the endoplasmic reticulum. The process is known as endoplasmic reticulum stress and is mediated by three main signaling pathways, the IRE1, ATF6, and PERK/eIF2α pathways (23, 24). PERK/eIF2α can upregulate the expression of activating transcription factor 4 (ATF4), which is an important molecule involved in ferroptosis. ATF4 can promote the expression of heat shock protein 5 (HSPA5) and further upregulate GPX4 to protect glioma cells from ferroptosis (25). Meanwhile, ATF4 can also promote the expression of cation transport regulator–like protein 1 (CHAC1) and degrade GSH in Burkitt lymphoma cells to induce ferroptosis (26). The dual effect of the PERK/eIF2α/ATF4 signaling on ferroptosis may be due to different pathological conditions; however, this regulatory pathway has not been fully studied in the context of CVDs.

Disease progression is often accompanied by the occurrence of multiple cell death modes. As organelles are structures that perform important biological functions in cells, determining their role in RCD is of interest. Clarifying the relationship between different cell death pathways and evaluating the incidence of specific death modes in the course of disease will help gain critical insights on the theoretical basis for the use of targeted therapies and the use of different combinations of drugs in preventing and treating CVDs.

## Connecting the Links Between Ferroptosis and Autophagy

**Figure 1:**
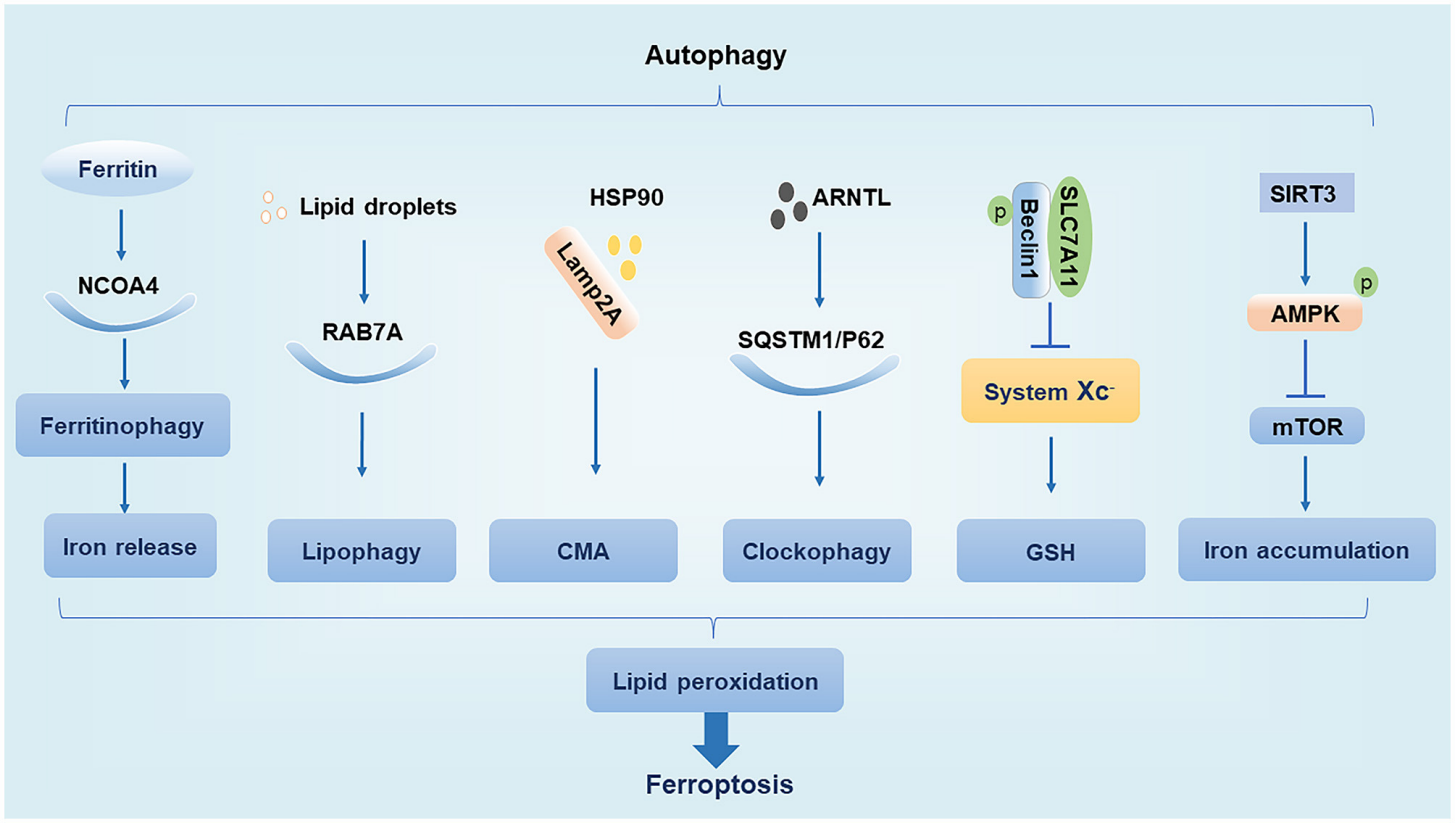
Relationship between autophagy and ferroptosis. NCOA4, nuclear receptor coactivator 4; Lamp2A, lysosome associated membrane protein 2A; HSP90, heat shock protein 90; AMPK, AMP-activated protein kinase.

The main function of autophagy is to remove the damaged organelles and proteins. Moderate levels of autophagy protect cells by digesting their senescent organelles and metabolic wastes; however, under excessive damage, autophagy can lead to apoptosis and necrosis, which may subsequently induce the development of various clinical diseases (27). The ferroptosis inducer erastin promotes the conversion of autophagy-related protein LC3 from LC3-I to LC3-II. In turn, autophagy regulates ferroptosis by affecting cellular iron homeostasis and ROS production (28). We summarize the interaction of regulatory molecules of autophagy and ferroptosis (Figure 1).

### Nuclear Receptor Coactivator 4

NCOA4 is a cargo receptor for autophagic ferritin degradation. It participates in ferritinophagy, thereby promoting the release of Fe^3+^ and increasing the intracellular concentration of free iron, which promotes ferroptosis. When cellular iron levels are high, NCOA4 is degraded by the ubiquitin E3 ligase HERC2, leading to reduced iron release. In contrast, when intracellular iron levels are low, the degradation of NCOA4 decreases and ferritinophagy increases, leading to increased iron release (19). Moderate levels of ferritinophagy can maintain the balance of intracellular iron, but excessive ferritinophagy can cause ferroptosis by increasing ROS accumulation (14, 28).

### RAB7A

Ras-related protein RAB7A is a member of the RAS oncogene family and is a cargo receptor required for lipophagy. Lipophagy can selectively recognize and degrade lipid droplets, increase free fatty acid production, promote lipid peroxidation, and induce ferroptosis. Studies have found that tumor protein D52 (TPD52) inhibits RSL3-induced ferroptosis by promoting lipid droplet storage, whereas knockdown of autophagy-related genes *ATG5* and *RAB7A* restricts RSL3-induced ferroptosis by inhibiting lipid droplet degradation (29). This suggests that the balance between lipid synthesis, storage, and degradation can regulate ferroptosis.

### Chaperone-Mediated Autophagy

CMA is a selective autophagy pathway for soluble cytosolic proteins that is mediated through molecular chaperones. Cytosolic heat shock cognate protein 70 (HSC70), also known as HSPA8, recognizes the KFERQ amino acid sequence to form a complex and bind to lysosome associated membrane protein 2A (Lamp2A), which is subsequently transported to the lysosomal membrane under HSC70 guidance where the target substrate is unfolded and translocated to the lysosome for degradation (30). Heat shock protein 90 (HSP90) binds to Lamp2A on the lysosomal membrane and activates CMA. It was found that GPX4 is degraded *in vitro* by HSP90-induced CMA to induce ferroptosis in cells (31). Interestingly, another study found that HSPA5 can in turn upregulate GPX4 to inhibit ferroptosis (25). Thus, various types of heat shock proteins appear to have different effects on ferroptosis.

### Ubiquitin-Binding Protein p62

The p62 protein, which is also known as sequestosome 1 (SQSTM1), is a selective autophagy connector protein that serves as a bridge between autophagy-related LC3 proteins and polyubiquitinated proteins. It then transports damaged proteins, mitochondria, and invading bacteria to autophagosomes for degradation (32). It has been determined that p62 can be used as a cargo receptor required for circadian clock autophagy and that it participates in autophagic degradation of ubiquitinated substrates. The term clockophagy is used to refer to the selective autophagy degradation of the core clock protein Aryl hydrocarbon receptor nuclear translocator-like protein 1 (ARNTL). Studies have found that ARNTL promotes the expression of Egl nine homolog 2 (EGLN2) *via* p62-mediated selective autophagic degradation and destroys the stability of hypoxia-inducible factor 1-alpha (HIF1A), which promotes lipid peroxidation and ferroptosis to exert antitumor effects (33). EGLN2, also known as prolyl hydroxylase 1 (PHD1), is an oxygen sensor that degrades HIF1A, which in turn inhibits lipid storage to promote ferroptosis (33, 34).

### Beclin1

Beclin1, the mammalian homolog of yeast autophagy-related gene 6 (ATG6), is a key protein necessary for the formation of autophagosomes (35). Studies have shown that phosphorylated Beclin1 interacts with SLC7A11, inhibits system Xc– activity, promotes GSH depletion, induces tumor cell lipid peroxidation, promotes ferroptosis, and increases antitumor efficacy (36, 37). Phosphorylated Beclin1 can also bind to phosphoinositol-3-kinase catalytic subunit type 3 (PIK3C3) to promote autophagy (38, 39). However, it is unclear whether phosphorylated Beclin1 preferentially interacts with SLC7A11 over that of PIK3C3 to promote ferroptosis.

### AMP-Activated Protein Kinase

AMPK is a serine/threonine-protein kinase whose activity is regulated by the AMP/ATP ratio, hypoxia, oxidative stress, and other factors. The mechanistic target of rapamycin (mTOR) is a negative regulator of autophagy, and AMPK enhances autophagy by inhibiting mTOR activity (40). In trophoblast cells treated with high glucose, SIRT3 has been shown to inhibit the activity of mTOR by enhancing the phosphorylation of AMPK to promote autophagy, thereby resulting in iron accumulation, lipid peroxidation, and induction of ferroptosis (41). However, the effect of AMPK activation on ferroptosis may have a dual effect as treatment of cells with glucose-free medium revealed that AMPK activation under conditions of energetic stress promotes the phosphorylation of acetyl coenzyme A carboxylase (ACC) to inhibit unsaturated fatty acid synthesis and ultimately inhibit ferroptosis (42). Thus, the regulation of ferroptosis by AMPK may depend on different stimulatory conditions, which needs to be further clarified in future studies.

In summary, several studies have investigated the connecting links between autophagy and ferroptosis, particularly in tumor cells. Ferroptosis promoted by autophagy can inhibit tumor cell growth, which is of great significance in the context of tumorigenesis and the development of resistance. However, it must be noted that in other diseases, autophagy is usually considered to be a protective phenomenon that can delay the development of diseases by inhibiting pyrolysis and apoptosis (21, 22). The role of autophagy-mediated ferroptosis in these diseases remains to be further studied. Owing to the contrary biological outcomes, there is a need to determine the relationship between autophagy and ferroptosis in non-tumor cells and the regulation of ferroptosis under different autophagic conditions.

## Mechanism of Ferroptosis

**Figure 2:**
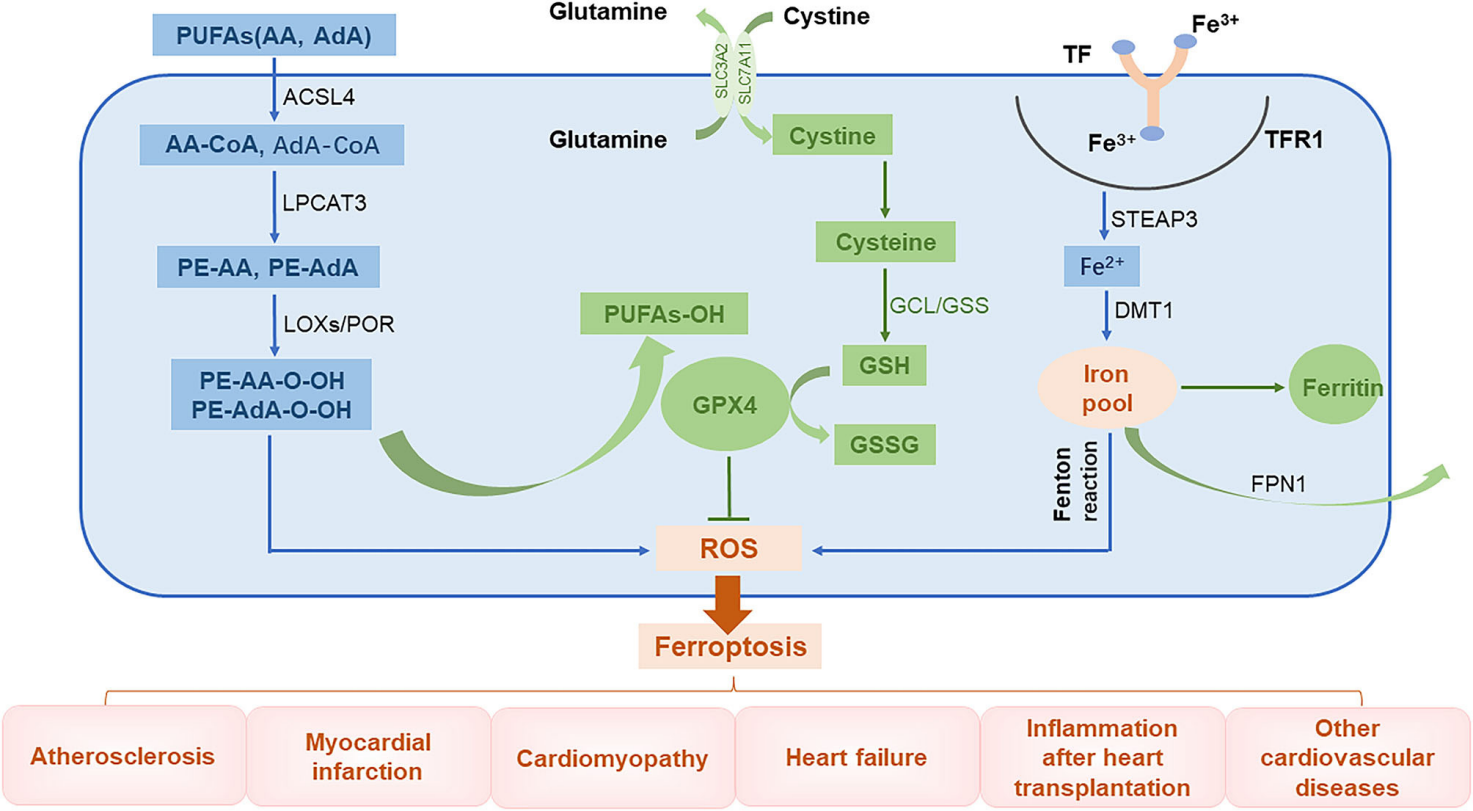
The role of ferroptosis in the pathophysiology of CVDs. PUFAs, polyunsaturated fatty acids; AA, arachidonic acid; AdA, adrenal acid; ACSL4, long-chain fatty acyl-CoA synthase 4; LPCAT3, lysolecithin acyltransferase 3; LOXs, lipoxygenases; POR, cytochrome P450 oxidoreductase; GCL, glutamate-cysteine ligase; GPX4, glutathione peroxidase 4; TF, transferrin; TFR1, transferrin receptor 1; Steap3, six-transmembrane epithelial antigen of prostate 3; DMT1, divalent metal transporter 1; FPN1, ferroportin 1.

The global prevalence of CVD-related mortality has been increasing each year. This makes it important to understand the basic molecular mechanisms underlying CVDs and to develop effective therapeutic drugs. Injury caused by oxidative stress is a key trigger for the development of CVDs (43). An imbalance between free radical production and scavenging is also encountered in ferroptosis, a phenomenon that is currently thought to be related to lipid, amino acid, and iron metabolism. For instance, when unsaturated fatty acids are oxidized into harmful lipid peroxides, they may damage the endothelium. In addition, excessive consumption of GSH will lead to increased accumulation of lipid peroxides, a phenomenon that affects cellular function. Furthermore, ROS generated by Fenton reaction can induce cell damage (44). In this review, we introduce the main mechanisms of abnormal lipid, glutamate acid, and iron metabolisms involved in inducing ferroptosis (Figure 2).

### Abnormal Lipid Metabolism

The lipid bilayer of cell membranes is essential for maintaining the integrity of membrane function. Cell membranes are mainly composed of lipids, proteins, and carbohydrates with polyunsaturated fatty acids (PUFAs) being an important component (45). Lipid peroxidation caused by interactions between unsaturated fatty acids and free radicals is the major cause of ferroptosis. Arachidonic acid (AA) and adrenal acid (AdA) are catalyzed by long-chain fatty acyl-CoA synthase 4 (ACSL4) to synthesize corresponding fatty acyl-coenzymes AA-CoA and AdA-CoA, respectively, which are involved in the synthesis of membrane phospholipids (46). These products are catalyzed by lysolecithin acyltransferase 3 (LPCAT3) through esterification reactions to form phosphatidylethanolamine PE-AA and PE-AdA, which are then oxidized by lipoxygenases (LOXs) or cytochrome P450 oxidoreductase (POR) into harmful lipid peroxidation products PE-AA-OOH and PE-AdA-OOH, respectively. Normally, these harmful substances are reduced by GPX4 to form non-toxic lipid alcohols; however, when the levels of GPX4 are low, these peroxide products excessively accumulate, leading to ferroptosis (47–49).

### Abnormal Glutamate Metabolism

Glutathione is an important antioxidant and free radical scavenger that is widely distributed in biological tissues and is catalytically produced from glutamate, cysteine, and glycine by glutamate-cysteine ligase (GCL) and glutathione synthase (GSS). Glutathione is an essential cofactor of glutathione peroxidase 4 (GPX4) and is required for its antioxidant activity (50). Glutathione has two forms, a reduced form (GSH) and an oxidized disulfide form (GSSG). Under the stimulation of free radicals and heavy metals, GPX4 converts GSH into GSSG and reduces lipid peroxides (L-OOHs) to its corresponding lipid alcohols (L-OHs). This limits the spread of lipid peroxidation in the membrane (8, 51). When GSH levels are reduced, ROS and lipid peroxides can accumulate, inducing ferroptosis (41, 50).

### Abnormal Iron Metabolism

Intracellular iron homeostasis depends on the dynamic balance between iron absorption, excretion, utilization, and storage. The distribution and content of iron in the body affects multiple physiological processes (52). Normally, the body's daily need for iron to maintain the balance of iron metabolism comes from the lysis of aging red blood cells and from ingested food. Gastric acids reduce Fe^3+^ in food to Fe^2+^, which is then absorbed in the duodenum and jejunum. The Fe^2+^ entering the body is then oxidized through the action of ceruloplasmin to Fe^3+^, which combines with transferrin (TF) on the cell membrane to form TF-Fe^3+^. This subsequently forms a complex with transferrin receptor 1 (TFR1). After being endocytosed into the cells, the iron is converted into Fe^2+^ by six-transmembrane epithelial antigen of prostate 3 (Steap3). Unstable Fe^2+^ is released into the cytoplasm by divalent metal transporter 1 (DMT1), also known as SLC11A2, and it then either enters the mitochondria or is directly utilized in the cytoplasm. The iron can also be stored in ferritin or secreted by ferroportin 1 (FPN1) (8, 53). Ferritin is composed of two subunits, a ferritin heavy chain (FTH1) and ferritin light chain (FTL) and is the most important iron storage protein in the cell. Iron that is not used in the cytoplasm or discharged from the cell is stored in ferritin (54). Iron levels are regulated by key proteins, such as TFR1, DMT1, and FTH1, to maintain intracellular iron homeostasis. Because iron has unpaired electrons, it can participate in redox reactions. In the Fenton reaction, Fe^2+^ and hydrogen peroxide (H_2_O_2_) react to generate hydroxyl radicals with strong oxidative capacity and Fe^3+^, which is then reduced by peroxide (O^2−^) back to Fe^2+^ to re-participate in the Fenton reaction. This cycle is called the Haber–Weiss reaction, where a large number of free hydroxyl radicals (a type of ROS) are produced (55). These free hydroxyl radicals can cause a series of clinical diseases (13, 56, 57).

Overall, ferroptosis is mainly caused by an imbalance between free radicals and the antioxidant systems, resulting in RCD. Most of the existing studies describe the mechanism of ferroptosis occurring in terms of lipid peroxide sources and degradation. The regulation of the above-mentioned metabolic molecules can clearly affect ferroptosis; however, there are many questions that need to be addressed by conducting further investigations. The first is whether other metal ions participate in ferroptosis, as noted before (9). The second question is that lipid peroxidation also occurs in other types of RCD, and whether more specific diagnostic methods could be developed for identifying ferroptosis.

## Ferroptosis is Involved in CVDs

As mentioned previously, ferroptosis plays various roles in the pathophysiology of CVDs. We elaborate on this in this section of the review.

### Atherosclerosis

AS is a pathological process characterized by lipid metabolism disorders. Endothelial injury, oxidative stress, inflammation, and immune dysfunction can each contribute to the development and progression of AS (58, 59). Clinically, coronary heart disease (CHD) is the most common type of organ lesion caused by atherosclerosis. Studies have shown that the iron content in tissues of individuals with AS is significantly increased compared to that of healthy arteries, and such iron overload promotes oxidative stress and inflammatory responses (60, 61). This suggests that ferroptosis may be involved in the development of AS. Researchers have found that *in vivo*, the ferroptosis inhibitor Fer-1 alleviates atherosclerotic lesions and lipid peroxidation induced by a high-fat diet in ApoE^−/−^ mice. Similarly, *in vitro* studies have demonstrated that Fer-1 can improve ferroptosis and endothelial dysfunction induced by ox-LDL and can delay the progression of AS (62). Therefore, inhibition of ferroptosis may be a new strategy for the treatment of CHD.

### Myocardial Infarction

MI is a common cause of death worldwide. In mice with MI, the levels of FTH1 in the infarct area decreased and free iron levels increased (63). Other studies have also found that the amount of GPX4 protein in MI tissue decreased compared with that of normal tissue and that the use of the GPX4 inhibitor RSL3 induces ferroptosis in H9c2 cells (64). These findings suggest that increased iron or decreased GPX4 can promote ferroptosis of cardiomyocytes and lead to MI. Currently, the most preferred treatment method for MI is recanalization being performed as soon as possible (65). Although the timely restoration of blood flow can partially save the damaged myocardium, prolongation of ischemia time can lead to damage, even when blood flow is restored, and this ischemia/reperfusion injury can increase mortality rates of patients with MI (66). In addition to calcium overload, oxidative stress, inflammation, and energy metabolism disorders, ferroptosis can also cause ischemia/reperfusion injury (67). Tang et al. found that ferroptosis mainly occurs in the reperfusion phase in a rat myocardial ischemia/reperfusion injury model, and further studies revealed that the use of DFO can reduce myocardial reperfusion injury (68). Surprisingly, the levels of the ferroptosis-associated proteins ACSL4 and GPX4 do not significantly change during the ischemic phase. The reason for this difference between the ischemia and reperfusion phases may be that large numbers of free radicals are produced during reperfusion to induce ferroptosis. In addition, it has been determined that the ferroptosis inhibitors liproxstatin 1 (Lip-1) and Fer-1 reduce ischemia/reperfusion injury by reducing ROS production (69, 70). Autophagy has also been found to promote ferroptosis during myocardial ischemia/reperfusion injury, leading to myocardial damage (71, 72). Therefore, actively preventing the occurrence of ferroptosis may become an important consideration after recanalization of patients suffering from MI.

### Cardiomyopathy

Cardiomyopathy is a group of heterogeneous myocardial diseases that causes mechanical and/or electrical dysfunction and occurs mostly due to hereditary causes. Such conditions often manifest from ventricular hypertrophy or dilation that may be confined to the heart itself or may be the result of systemic diseases. The outcome of cardiomyopathy may lead to cardiac arrest or progressive heart failure (73). The accumulation of iron in the myocardium can cause iron-overload cardiomyopathy, which usually presents in the early stages as diastolic dysfunction. As the disease progresses, the heart may enlarge and contraction may decrease (74). Ferroptosis is associated with many types of cardiomyopathies. Although there are differences in the pathogenesis of the different types, it is ultimately due to an imbalance in the production and scavenging of free radicals that causes an increase in lipid peroxides.

#### Dox-Induced Cardiomyopathy

Studies have found that the chemotherapeutic drug DOX induces DNA double-strand breaks in cardiomyocytes and increases Fe^2+^ and Ca^2+^ levels in mitochondria. The resulting increase in ROS and iron may be the main cause of DOX-induced ferroptosis, leading to cardiomyopathy (75–80). As mitochondria are the main organelles of DOX-induced myocardial damage, the mitochondrial antioxidant MitoTEMPO can be used to eliminate DOX-induced lipid peroxidation and ferroptosis (5). Iron chelators targeting Fe^2+^ may also be an ideal method to treat myocardial damage caused by DOX (81).

#### Hypertrophic Cardiomyopathy

Studies have also shown that high-iron feeding mice (FthMCK/MCK and FthMyh6/Myh6) that knock out the gene encoding the ferritin heavy chain (Ferritin H, Fth) of cardiomyocytes are prone to induce hypertrophic cardiomyopathy (82). Further studies suggest that the deficiency of GSH due to the downregulation of SLC7A11 is the main mechanism that induces ferroptosis in cardiomyocytes (82).

### Heart Failure

HF is the terminal stage of CVD, but the timely prevention of cardiomyocyte hypertrophy can maintain heart function and delay progression. Ferroptosis has been observed in mice with heart failure (83). Toll-like receptor 4 (TLR4) binds to NADPH oxidase 4 (NOX4), which promotes the production of superoxide anion and H_2_O_2_ and induces ferroptosis in cardiomyocytes. Therefore, blocking the TLR4-NOX4 signaling pathway may serve as a potential therapeutic strategy for treating patients with HF by inhibiting ferroptosis (84). It is strongly believed that iron is a cofactor involved in the energy metabolism of cardiomyocytes (85). Iron supplementation is recommended as it can improve the cardiac function in some patients with chronic HF who have iron deficiency (85). Therefore, determining the mechanism of ferroptosis will also be beneficial for the treatment of patients with HF.

### Inflammation After Heart Transplantation

Heart transplantation is the most effective method for treating patients with end-stage heart disease. However, the restoration of coronary blood flow after transplantation may result in aseptic inflammation and reduce the success rate of transplantation (86). After mice with heart transplantation, damage-associated molecular patterns (DAMPs) released from injured tissue promote neutrophil recruitment and infiltration via the TLR4/Trif signaling pathway. Fer-1 decreases the number of neutrophils adhering to blood vessels and inhibits inflammation. These findings demonstrate that inhibition of ferroptosis may be a viable therapeutic strategy for treating heart transplant recipients by reducing both inflammation and release of DAMPs (86).

### Other CVDs

Myocardial damage caused by sepsis may also be related to ferroptosis. In the myocardium of mice with sepsis-induced myocardial damage, the expression of GPX4 and GSH are reduced. Meanwhile, iron concentration and transferrin receptor levels increase and ferritin levels decrease during sepsis. According to Wang et al., these changes may be related to heme degradation caused by heme oxygenase-1 (HO-1) (87). It had been previously suggested that HO-1 has anti-cancer, anti-inflammatory, anti-apoptotic, and anti-oxidation effects (88). In the study by Wang et al., dexmedetomidine (DEX) was found to exert its protective effects possibly by reducing the expression of HO-1 and decreasing iron overload (87). HO-1 may exhibit dual roles in the regulation of ferroptosis under different pathological conditions. However, the role of HO-1 requires further study.

## Regulation of Ferroptosis for the Treatment of CVDs

As ferroptosis plays a detrimental role in CVD, intervening in this process may help prevent the development and progression of CVDs. Existing ferroptosis inhibitors, including clinically approved drugs and small-molecule drugs currently in the research phase of development, mainly eliminate free radicals, reduce free iron levels, or inhibit lipid peroxidation. This section of the review will focus on the above noted aspects of inhibiting ferroptosis (Table 1).

**Table 1:** Strategies to target ferroptosis in cardiovascular diseases.

**Therapeutic application**	**Mechanisms**	**Diseases**	**References**
Appropriate low-iron diet	—	—	(82)
**CLINICAL DRUGS**			
DFO	—	Ischemia/reperfusion	(68)
Dex	Reduces the expression of HO-1, iron overload, and inflammatory factors; Enhances the expression of GPX4	Septic heart injury	(87)
Puerarin	Induces the production of FTH1 and GPX4; Reduces the production of ROS and NOX4	Heart failure	(89)
Baicalin	Inhibits ACSL4	Ischemia/reperfusion;	(90)
AsIV	Activates Nrf2	Cardiac hypertrophy Adriamycin-induced cardiac ferroptosis	(91, 92)
C3G	Decreases Fe^2+^; Downregulates TFR1; Promotes GPX4 and FTH1	Ischemia/reperfusion	(93)
**ROS INHIBITORS**			
Fer-1	Reduces the production of ROS	Diabetes myocardial; Ischemia/reperfusion	(70)
Lip-1	Reduces the production of mitochondrial ROS; Restores the level of GPX4	Ischemia/reperfusion	(69)
Mito–TEMPO	Eliminates peroxides	Cardiomyopathy	(5)
**KEY TARGETS FOR INHIBITING FERROPTOSIS**			
GPX4	Inhibits lipid peroxidation	Ischemia/reperfusion; Myocardial infarction; Cardiomyopathy	(64, 69, 81)
SLC7A11	Increases GSH	Cardiomyopathy; Heart failure	(82, 88)
NRF2	Activates NQO1, HO-1, and FTH1; Increases SLC7A11	—	(94, 95)
FSP1	Reduces CoQ Generate lipophilic free radical trapping antioxidants (RTA)	—	(96)
DHODH	Reduces CoQ to CoQH2	—	(97)
Non-coding RNA	miR-23a-3p inhibits DMT1	AMI	(98)

*DFO, Deferoxamine; Dex, Dexmedetomidine; Met, Metformin; AsIV, Astragaloside IV; C3G, Cyanidin-3-glucoside; Fer-1, Ferrostatin-1; Lip-1, Liproxstatin-1; HSF1, Heat shock transcription factor 1; FSP1, Ferroptosis suppressor protein 1; DHODH, Dihydroorotate dehydrogenase; NRF2, Nuclear factor E2-related factor 2; AMI, Acute myocardial infarction*.

### Appropriate Low-Iron Diet

The normal iron content in the body is 3–5 g and an insufficient supply of iron may impair the synthesis of ferritin at levels required for normal cell physiology, leading to a series of adverse consequences (99). In contrast, iron overload may result in the production of large amounts of ROS and induce lipid peroxidation and ferroptosis (82). Therefore, appropriate low-iron diets with consideration of minimum requirements may help prevent CVDs caused by ferroptosis.

### Clinical Drugs

DFO is a high-affinity iron chelator that reduces intracellular iron and has been approved by the US Food and Drug Administration for the treatment of iron overload. In rats with ischemia/reperfusion injury, DFO can reduce lipid peroxides in cardiac tissue and attenuated myocardial injury (68).

DEX is an α_2_ adrenergic receptor agonist. Studies have shown that pretreatment with DEX can reduce myocardial ischemia/reperfusion injury by inhibiting the release of inflammatory factors IL-1β, IL-6, and TNF-α through the TLR4-MyD88-NF-κB signaling pathway (100). DEX can inhibit apoptosis and oxidative stress by activating the phosphatidylinositide 3-kinase/protein kinase B (PI3K/Akt) signaling pathway, thereby protecting diabetic rats from ischemia/reperfusion injury (101). It also has anti-inflammatory, antioxidant, and protective effects on ischemia/reperfusion organs. Recently, DEX was found to reduce the expression of HO-1, reduce iron overload and inflammatory factors, and enhance the expression of GPX4, thereby inhibiting ferroptosis and alleviating myocardial cell damage induced by sepsis (87).

Puerarin is an isoflavone compound extracted from plants that has been used in traditional Chinese medicine. Puerarin has been shown to prevent ischemia/reperfusion injury by inhibiting pyrolysis (102), reducing the area of MI, improving diastolic cardiac function (103), and preventing myocardial fibrosis (104). Puerarin has been approved by the China Food and Drug Administration as a clinical treatment drug for CVDs (105). Recent studies by Liu et al. have found that puerarin can also improve cardiac function in rats with HF by reducing iron content and lipid peroxidation, thereby inhibiting ferroptosis (89). Liu et al. found that treatment of H9c2 cells with erastin or isoproterenol can cause ferroptosis. Puerarin was shown to inhibit ferroptosis in cardiomyocytes by inducing the production of FTH1 and GPX4 and reducing the production of ROS and NOX4, thus improving the cardiac function of rats with HF (68).

Baicalin is a natural flavonoid compound isolated from the root of the plant *Scutellaria baicalensis* Georgi and has anti-inflammatory, anti-viral, anti-cancer, antioxidant, and other pharmacological effects (106). Studies have shown that baicalin has a protective effect on myocardial ischemia/reperfusion, reducing myocardial apoptosis and inflammation by activating the PI3K/AKT pathway (107). Furthermore, baicalin protects the microvascular endothelial cells in the heart of ischemia/reperfusion rats by activating the PI3K-AKT-eNOS pathway (108). Fan et al. found that baicalin can also play a cardioprotective role by inhibiting ferroptosis induced by ACSL4 (90). ACSL4 is a key enzyme in fatty acid metabolism and synthesizes AA and AdA into AA-CoA and AdA-CoA, respectively, which are involved in membrane phospholipid synthesis and promote ferroptosis (109).

Astragaloside IV (AsIV) is extracted from *Astragalus* spp. and is used in traditional Chinese medicine. It protects the myocardium by inhibiting myocardial cell hypertrophy, apoptosis, and fibrosis. Studies have confirmed that Nrf2 may be a key molecule targeted by AsIV for it to exert its protective effect. Upregulation of Nrf2 can inhibit cardiac hypertrophy in an abdominal aortic constriction model of chronic HF (91). Furthermore, the activation of Nrf2 signaling and the expression of GPX4 can inhibit ferroptosis, thus further preventing adriamycin-induced cardiac fibrosis (92).

Cyanidin-3-glucoside (C3G) is a member of the anthocyanin family, is found in a wide variety of vegetables and fruits, and has anti-inflammatory and antioxidant effects. C3G can reduce DOX-induced cardiotoxicity in mice and prevent cardiac hypertrophy and diastolic cardiac dysfunction (110, 111). Experiments have shown that C3G alleviates oxidative stress in rats with ischemia/reperfusion injury and in H9C2 cells with oxygen-glucose deprivation/reoxygenation (OGD/R). Treatment with C3G leads to decreased Fe^2+^ content, downregulation of TFR1 expression, and promotion of GPX4 and FTH1 expressions. Therefore, C3G is a potential drug that may be used to protect the myocardium from ischemia/reperfusion injury (93).

### ROS Inhibitor

Fer-1 is a specific inhibitor of ferroptosis that was identified through high-throughput screening of small-molecule libraries (112). Fer-1 can inhibit the accumulation of lipid ROS and reduce cell ferroptosis induced by erastin or RSL3. It has also been has found that Fer-1 can inhibit ferroptosis and improve DOX-induced myocardial damage in CVDs and alleviate myocardial ischemia/reperfusion injury in diabetic rats (5, 70).

Lip-1 has a mechanism of action similar to that of Fer-1. Without affecting the Ca^2+^-induced mitochondrial permeability transition pore opening, Lip-1 reduces the production of mitochondrial ROS, restores GPX4 levels by downregulating the level of VDAC1 (without affecting VDAC2/3) and oligomerization to protect myocardial mitochondria from ischemia/reperfusion injury (69). VDACs are located in the outer membrane of mitochondria, regulate mitochondrial metabolism and cell-signal transduction, and are transmembrane channels used for the transport of ions and metabolites (113). After erastin acts on VDACs, permeability of the outer mitochondria membrane increases, membrane ion channels open, and intracellular homeostasis becomes disturbed, leading to mitochondrial metabolism and oxidation dysfunction, increased ROS production, enhanced lipid peroxidation, and the subsequent induction of cell ferroptosis (15).

Mito-TEMPO is a cell-permeable mitochondria-targeted antioxidant that reduces the accumulation of superoxides (114). Mito-TEMPO can also reduce cell apoptosis by reducing Ca^2+^ release (115). In recent years, Mito-TEMPO has been shown to effectively inhibit DOX-induced ferroptosis of cardiomyocytes by eliminating lipid peroxidation in mitochondria, which is a cardioprotective effect similar to that of Fer-1 (5).

### Key Targets for Inhibiting Ferroptosis

GPX4 can reduce lipid peroxides (L-OOHs) to lipid alcohols (L-OH), which inhibit the spread of lipid hydroperoxides and protect cells from damage. When levels of GPX4 is reduced, ferroptosis is promoted and ischemia/reperfusion injury (69), MI (64), and cardiomyopathy (81) can be induced.

SLC7A11, also known as xCT, is a multichannel transmembrane protein that serves as a component of the cystine/glutamate reverse transporter, system Xc–. System Xc– is an antiporter, consisting of SLC7A11 as the light chain subunit and SLC3A2 as the heavy chain subunit. System Xc– transfers intracellular glutamate to the outside of cells and extracellular cystine into the cells. The cystine entering the cell is converted to cysteine by a reduction reaction and then used in the synthesis of GSH, which protects the cells from oxidative stress damage. The transport direction of system Xc– is driven by substrate concentrations. When the concentration of extracellular glutamate is high, the uptake of cystine by system Xc– is inhibited, which leads to a reduction in the synthesis of intracellular GSH and decreases the antioxidant capacity of the cells, eventually leading to the accumulation of lipid ROS and the induction of ferroptosis (50, 82).

Nuclear factor E2-related factor 2 (NRF2) is a transcriptional activator that plays an important role in antioxidant responses and regulates ferroptosis through the p62-KEAP1-NRF2 signaling pathway (116). Under normal physiological conditions, NRF2 and KEAP1 bind one another and are anchored in the cytoplasm in an inactive state. When the cell is under oxidative stress, the conformation of KEAP1 changes, KEAP1 dissociates from NRF2, and NRF2 enters the nucleus where it recognizes components of antioxidant reactions and regulates the expression of genes related to oxidative damage, thereby playing an antioxidant role. The structure of p62 includes an STGE sequence that can bind to the Kelch domain of KEAP1, which reduces the stability of the KEAP1/NRF2 complex causing NRF2 to dissociate from the complex and become activated (117). This further activates the transcription of NQO1, HO-1, and FTH1, or increases the expression of SLC7A11 to inhibit ferroptosis (94, 95). While these results demonstrate that HO-1 has a protective effect, a previous study found that HO-1 can degrade heme and promote ferroptosis (87). Therefore, HO-1 may have a dual role in ferroptosis. The mechanisms of these different models require further investigation.

At least three defense mechanisms have been found to inhibit ferroptosis. These include (1) GPX4 using reduced GSH to eliminate lipid peroxidation and inhibiting ferroptosis; (2) Ferroptosis suppressor protein 1 (FSP1), also known as AIFM2, acting as an oxidoreductase that primarily reduces CoQ to a lipophilic radical, trapping antioxidant (RTA) at the plasma membrane and preventing the spread of lipid peroxidation (96); and (3) Dihydroorotate dehydrogenase (DHODH), an enzyme located on the outer surface of mitochondrial intima, reducing CoQ to CoQH2 while oxidizing dihydroorotate (DHO) to orotate (OA) and cooperating with GPX4 to inhibit lipid peroxidation and ferroptosis in mitochondria (97). The first mechanism has been verified in CVDs; it remains to be determined whether the latter two defense mechanisms also play protective roles in CVDs.

### Non-coding RNA

ncRNAs are classified as long non-coding RNA (lncRNA), microRNA (miRNA), and small interfering RNA (siRNA) according to their length. Recent studies have shown that ncRNAs are differentially expressed in CVDs and play an important role in CVD development (118). A previous study found that exosomes derived from human umbilical cord mesenchymal stem cells (HUCMSCs) can suppress DMT1 expression through the delivery of miR-23a-3p, thereby inhibiting ferroptosis and attenuating myocardial injury (98). DMT1 promotes iron metabolism by transporting Fe^2+^, and the knockout of DMT1 significantly inhibits ferroptosis (98). This suggests ncRNAs may be an important factor in the intervention of ferroptosis and that exosomes may serve as transport carriers to deliver the ncRNAs (119).

In the cardiovascular field, existing research has shown that it is possible to suppress the occurrence of ferroptosis and prevent the development of the disease through various mechanisms. However, most of the experiments remain at the animal or cell level, so the application of these findings to clinical treatment requires additional detailed analysis and appropriate clinical trials.

## Conclusions and Prospects

Since the discovery of ferroptosis as a new type of RCD, in-depth biological research has provided new ways to explore its occurrence and role in a variety of diseases. A growing number of studies have shown that lipid peroxidation-induced ferroptosis is an important pathogenetic mechanism of CVD development. Although, this discovery poses a challenge to the treatment of CVDs, it also provides a new therapeutic target. Current research continues to reveal details regarding the mechanism of action of ferroptosis in a variety of diseases and also raises many questions that are worthy of researchers' consideration. First, further research is needed on the interaction between ferroptosis and other modes of RCD. The key ferroptosis proteins P53, GPX4, and SLC7A11 are also involved in other modes of death. When these proteins are changed, we need to be able to distinguish their interactions among the different types of RCD through experimental means. Second, further insight into the manifestation of ferroptosis is needed. For instance, lipid peroxidation is also involved in other forms of RCD and may be only an intermediate link in ferroptosis. Third, defining the threshold peroxidation reaction is essential. Oxygen-free radicals in humans and other species are necessary for metabolism and can also induce ferroptosis. The threshold of oxygen free radicals required for inducing ferroptosis needs to be further studied. Fourth, ways need to be determined to regulate ferroptosis. As there are different regulatory pathways for ferroptosis, it is necessary to clarify whether there are intersecting points between each pathway and the final executive molecule. Finally, we need to be able to ultimately control the “double-edged sword” of ferroptosis. Their effects must be carefully balanced while developing or administering drugs and other therapeutic interventions.

The significance of the basic research regarding ferroptosis and CVD lies in its ultimate clinical relevance. Experimental research needs to continuously improve on available strategies before it can provide hope for new treatment options for patients suffering from CVDs. We have a reason to believe that with the advancement of science, research on ferroptosis will help provide a solid theoretical basis on the occurrence and development of CVDs. Furthermore, we believe that the specific targeting of ferroptosis will emerge as an important means of treating CVD.

## Author Contributions

ZC and YY researched the article and wrote the manuscript. CQ, JL, LL, and JW reviewed and edited the manuscript before submission. All authors provided substantial contribution to the discussion of content.

## Funding

This work was supported by Development and Reform Commission of Jilin Province (2020C036-3) and the Science and Technology Development of Jilin Province (20200301003RQ).

## Conflict of Interest

The authors declare that the research was conducted in the absence of any commercial or financial relationships that could be construed as a potential conflict of interest.

## Publisher's Note

All claims expressed in this article are solely those of the authors and do not necessarily represent those of their affiliated organizations, or those of the publisher, the editors and the reviewers. Any product that may be evaluated in this article, or claim that may be made by its manufacturer, is not guaranteed or endorsed by the publisher.
